# Comparative Study of the Efficacy of Anti-CGRP mAbs on Migraineurs: Analysis of the First Year of Therapy, 1-Month Suspension Period, and Reprisal

**DOI:** 10.3390/jcm12237329

**Published:** 2023-11-26

**Authors:** Yan Tereshko, Simone Dal Bello, Sara Pez, Enrico Belgrado, Christian Lettieri, Bruno Hector Ercole, Giulia Cellante, Caterina Del Regno, Giuseppe Sportelli, Giovanni Ermanis, Salvatore Versace, Giovanni Merlino, Gian Luigi Gigli, Mariarosaria Valente

**Affiliations:** 1 Clinical Neurology Unit, Udine University Hospital, Piazzale Santa Maria della Misericordia 15, 33100 Udine, Italy; 2 Department of Medicine (DAME), University of Udine, Via Colugna 50, 33100 Udine, Italy; 3 Neurology Unit, Udine University Hospital, Piazzale Santa Maria della Misericordia 15, 33100 Udine, Italy

**Keywords:** anti-CGRP mAbs, erenumab, galcanezumab, fremanezumab, chronic migraine, high-frequency episodic migraine

## Abstract

**Background:** Few studies compare the clinical effectiveness of the three anti-CGRP mAbs. Moreover, no studies compare their efficacy during suspension and reprisal. Our study aimed to compare the efficacy of migraine frequency, intensity, and symptomatic medication intake during the first year of therapy, a 1-month suspension period, and a 3-month drug reprisal. **Methods:** A total of 160 migraineurs (chronic and high-frequency episodic) were treated with anti-CGRP mAbs (49 with fremanezumab, 55 with erenumab, and 55 with galcanezumab) for 12 months. They discontinued the therapy for 1 month and then reprised the therapy. In the three groups, we analyzed and compared the migraine days per month, migraine intensity, and symptomatic medication intake per month at baseline, 3-month, 6-month, and 12-month follow-up. We also compared these variables during the 1-month suspension and 3 months after the reprisal of the therapy. We compared the data and evaluated the response rate (>50% reduction in migraine days per month) at different follow-ups. This comparison was also performed separately for chronic and high-frequency episodic migraineurs. **Results:** There was no statistical difference in monthly migraine days, intensity, or symptomatic medication intake per month at the different follow-ups. Moreover, there was no difference in the response rate overall. However, in chronic migraineurs treated with galcanezumab, the response rate was higher during the 1-month suspension when compared to fremanezumab and erenumab. In high-frequency episodic migraineurs, fremanezumab had a higher response rate at 12-month follow-up when compared to galcanezumab and erenumab. **Conclusions:** In our study, the three anti-CGRP mAbs presented a similar response, with no significant differences, during the first year of therapy, the suspension period, and 3 months after the drug reprisal. The response rate during the 1-month suspension period in chronic migraineurs may be higher with galcanezumab.

## 1. Introduction

Calcitonin gene-related peptide (CGRP) is a pivotal player in the intricate pathology of migraines [1,2]. Recently, a groundbreaking shift in migraine prophylactic therapies has transpired with the advent of novel monoclonal antibodies targeting CGRP or its receptor (anti-CGRP mAbs). Erenumab exerts its mechanism by blocking the CGRP receptor, while fremanezumab and galcanezumab directly engage with the peptide [3]. CGRP is a molecule that plays an important role in migraine. These preventive therapies have been developed to antagonize its action, and it has been demonstrated that the serum levels of this molecule increase during migraine attacks and decrease with the administration of triptans [4,5]. Moreover, intravenous infusion of CGRP can induce migraine attacks [6].

Pivotal studies underscore all three antibodies’ remarkable efficacy in diminishing monthly migraine days and alleviating pain intensity, surpassing the outcomes observed with placebos. Notably, their exceptional safety and tolerability profile is attributed to their highly selective mechanism of action [7,8,9]. These clinical results were confirmed in real-life studies, highlighting a significant benefit on patients’ quality of life [10,11,12].

Only a few studies directly compare the clinical efficacy of the three anti-CGRP monoclonal antibodies [13]. Furthermore, there is an absence of studies assessing the impact of discontinuation and subsequent resumption of the three distinct monoclonal drugs on migraine characteristics. Indeed, after one year of treatment with anti-CGRP or CGRP-receptor monoclonal antibodies, the AIFA (Italian Medicines Agency, Rome, Italy) imposes a one-month treatment withdrawal. This suspension is to assess whether, one month later, the indication for monoclonal antibody use persists (as a matter of cost of public spending). The antibody to choose in each case depends mainly on the patient’s preference for the device or on the physician’s choice without a clear clinical indication. Comparative data on the efficacy and discontinuation period of the three antibodies help define more precisely which CGRP mAb should be used in clinical practice and for personalized treatment.

For this reason, the present study aimed to directly compare the efficacy of three different anti-CGRP mAbs (erenumab, fremanezumab, and galcanezumab) by assessing the reduction in migraine days and pain intensity in the first year of treatment. We also aimed to compare the frequency and intensity of migraine attacks during cessation and after resumption of the three preventive therapies.

## 2. Methods

### 2.1. Study Design and Participants

This was a retrospective longitudinal single-center study with prospectively collected data from 205 patients aged > 18 years with chronic and high-frequency episodic migraine according to the ICHD-3 criteria [14] and treated with anti-CGRP mAbs (erenumab 140 mg per month, fremanezumab 225 mg per month, or galcanezumab 120 mg per month + 240 mg for the loading dose during the first administration) in our tertiary headache center from January 2020 to May 2023. The decision of each treatment was based on the clinician’s discretion. We included in our analysis only the patients who concluded a one-year cycle of therapy with anti-CGRP mAbs, and suspended the therapy for 1 month (according to Italian Medicines Agency guidelines; this 1-month suspension period was introduced to assess the patients that did not require the anti-CGRP mAbs anymore for migraine prophylaxis, to reduce the expenses on the Italian national health-care system), and then restarted the therapy for at least 3 months. All the patients included needed to have in their headache diary details of migraine intensity, symptomatic medication intake, and migraine frequency (days per month). The presence of concomitant oral preventive therapy was allowed if it was taken for at least 3 months before the initiation of preventive treatment with anti-CGRP mAbs and was not modified during the observation period. The presence of a concomitant medication overuse headache (MOH) was allowed (more than 15 days of headache per month in a patient with a preexisting headache as a consequence of overuse of symptomatic medication that presents for more than three months; the intake of symptomatic medications needs to be >10 or >15 days per month depending on the medication) [14]. The patients were evaluated at baseline (T0) and then at 3-month (T1), 6-month (T2), and 12-month follow-up (T3). They were also evaluated at the end of the month of drug suspension (T4) and 3 months after the drug restart (T5). The entire follow-up period was 16 months. The patients that filled the criteria for the statistical analysis were 160. Among the 45 excluded patients, 11 did not restart the therapy after the suspension, 13 patients were under treatment but had not finished the first year, 2 patients shifted to another anti-CGRP mAb, 7 patients stopped the therapy due to lack of efficacy, 3 stopped due to adverse events (2 patients had a significant local allergic reaction, 1 had consistent constipation), and 12 patients had incomplete data.

### 2.2. Ethics

This study was conducted following the Declaration of Helsinki and approved by the Institutional Review Board of the University of Udine (IRB-DAME; RIF. Prot IRB: 175/2023; Tit III cl 13 fasc. 5/2023). All patients gave written consent for the treatment and their clinical data to be used for research purposes.

### 2.3. Endpoint

To compare the efficacy of the three anti-CGRP mAbs (erenumab, galcanezumab, fremanezumab) during the first year of treatment, the 1-month suspension, and 3 months after the reprisal, based on migraine intensity (NRS 1–10), frequency (migraine days per month), and symptomatic medication intake (number per month). The comparison was also performed for chronic and high-frequency episodic migraineurs.

### 2.4. Statistical Analysis

The descriptive analysis of the sample was performed using means ± SD for continuous variables and percentages for categorical variables. A Shapiro–Wilk test was used to assess the normal distribution of data. Baseline comparison was performed using ANOVA test for quantitative variables and chi-squared test with a 3 × 2 contingency table for qualitative variables. The calculated power of the study is 96% (G*power 3.1, ANOVA repeated measure, within–between interaction). We set an effect size of 0.25, an *α*-error of 0.05, three groups, six measurements, 160 subjects, and a correlation among repeated measures of 0.5; we calculated the power of the study as 0.96. Repeated-measure ANOVA for the three anti-CGRP mAbs was performed to investigate the changes and the differences between the three therapies in migraine intensity (NRS), migraine frequency (days of migraine per month), and symptomatic medication intake (number per month) at baseline (T0) and at 3-month (T1), 6-month (T2), 12-month (T3), 1-month suspension period (T4), and 3-month post-suspension follow-up (T5). Because Mauchly’s test of sphericity was significant, we used the Greenhouse–Geisser correction. The Bonferroni post hoc test was used to compare the means at different follow-up times. A chi-squared test with a 3 × 2 contingency table was performed to compare the rate of responders (>50% reduction in migraine days) during the follow-up phases. All analyses used Stata/SE (version 15.1, StataCorp, College Station, TX, USA) for Mac OS. All 2-tailed statistical significance levels were set at *p* < 0.05.

## 3. Results

A total of 160 patients treated with anti-CGRP mAbs were examined. Of these, 49 were treated with fremanezumab, 56 with erenumab, and 55 with galcanezumab. The detailed demographics, characteristics, and features of migraine in each group are shown in Table 1. The fremanezumab group had higher frequency and intensity when compared to the other two groups. Considering only chronic migraineurs, the fremanezumab group had higher frequency when compared to the erenumab group, and the galcanezumab had the lowest intensity compared to the other two groups. In the high-frequency episodic migraineurs, erenumab had a higher frequency compared to the galcanezumab group.

We analyzed the migraine frequency (days per month), intensity (NRS), and the number of symptomatic medication intake (number per month) at baseline (T0) and 3-month (T1), 6-month (T2), 12-month follow-up (T3), after 1 month of drug suspension (T4), and at the 3-month reprisal of the therapy (T5).

**Figure 1:**
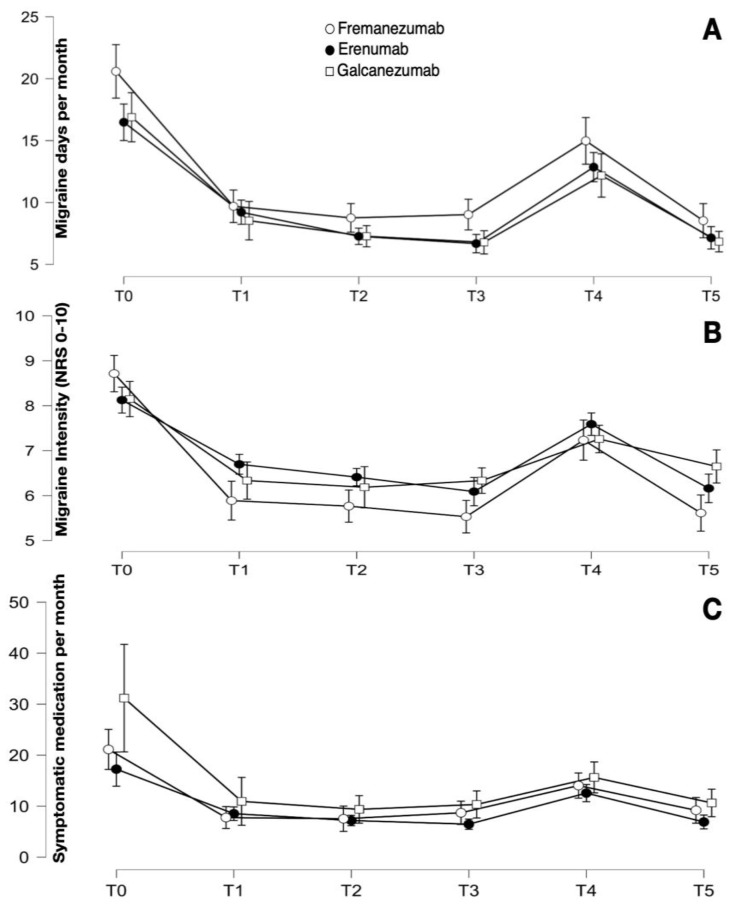
(**A**) Number of mean migraine days per month at the different phases of follow-up in all the patients divided per anti-CGRP mAb. (**B**,**C**) Mean intensity and mean number of symptomatic medication intake per month, respectively. The bars represent the confidence interval.

There was a statistically significant reduction in migraine frequency (F_3.138, 492.743_ = 124.537; *p* < 0.001), intensity (F_4.250, 662.927_ = 87.223; *p* < 0.001), and symptomatic medication intake (F_1.781, 257.741_ = 32.334; *p* < 0.001). Moreover, there was no statistical difference between the three anti-CGRP mAbs in terms of frequency (F_2.157_ = 2.125; *p* = 0.123), intensity (F_2.156_ = 1.472; *p* = 0.233), or symptomatic medication intake (F_2.150_ = 2.906; *p* = 0.058). See Table 2 for detailed data and Figure 1.

The migraine frequency improved from baseline to T1 (*p* < 0.001 in each group), T2 (*p* < 0.001 in each group), T3 (*p* < 0.001 in each group), T4 (*p* < 0.001 in the galcanezumab and fremanezumab groups; 0.010 in the erenumab group), and T5 (*p* < 0.001 in each group). Moreover, there was a worsening in migraine frequency in the three anti-CGRP mAbs groups during T4, and it was statistically significant when compared to T3 (*p* < 0.001 in all three groups) and T5 (*p* < 0.001 in all three groups). There was no difference considering T3 vs. T5 (*p* = 1.000), T1 vs. T2 (*p* = 1.000), T1 vs. T3 (*p* = 1.000 for fremanezumab and galcanezumab, 0.455 for erenumab), or T2 vs. T3 (*p* = 1.000).

Migraine intensity significantly improved at T1 (*p* < 0.001 in the three groups), T2 (*p* < 0.001 in the three groups), T3 (*p* < 0.001 in the three groups), and T5 (*p* < 0.001 in the three groups) when compared to T0; in the erenumab group, there was no difference between T0 and T4 (*p* = 1.000). The difference remained statistically significant in the galcanezumab and erenumab groups despite the worsening at T4 (*p* = 0.026 galcanezumab, <0.001 fremanezumab). Moreover, there was no difference between T3 and T5 in each group (*p* = 1.000); in all groups, the intensity was significantly lower at T3 than in T4 (*p* < 0.001 in the erenumab and fremanezumab groups; 0.015 in the galcanezumab group). There was significant improvement in intensity at T5 when compared to T4 for the fremanezumab group and erenumab (*p* < 0.001) groups, but not for the galcanezumab group (*p* = 0.868). There were no differences between T1 vs. T2 (*p* = 1.000), T1 vs. T3 (*p* = 1.000 for fremanezumab and galcanezumab, 0.810 for erenumab) and T2 vs. T3 (*p* = 1.000).

There was a significant reduction in symptomatic medication intake at T1 (*p* < 0.001 for fremanezumab and galcanezumab), T2 (*p* < 0.001 in the galcanezumab and fremanezumab groups; 0.008 for erenumab), T3 (*p* < 0.001 in the galcanezumab and fremanezumab groups; 0.002 for erenumab), and T5 (*p* = 0.001 for fremanezumab; *p* = 0.005 for erenumab; *p* < 0.001 for galcanezumab) when compared to T0. Moreover, in the erenumab group, the medication intake reduction was not statistically significant when comparing T0 to T1 (*p* = 0.063) and gained significance from the T2. In the fremanezumab and galcanezumab groups, the reduction in terms of symptomatic medication intake was statistically significant from T1.

The symptomatic medication intake was not statistically different when T4 was compared to T0 in the fremanezumab and erenumab groups (fremanezumab *p* = 0.888; erenumab *p* = 1.000). In the galcanezumab group, symptomatic intake worsened T4; however, the number was statistically lower than T0 (*p* < 0.001). There were no differences when comparing T3 vs. T4 (*p* = 1.000 for each group), T4 vs. T5 (*p* = 1.000 for each group), and T3 vs. T5 (*p* = 1.000 for each group). There were no differences between T1 vs. T2 (*p* = 1.000), T1 vs. T3 (*p* = 1.000), and T2 vs. T3 (*p* = 1.000).

### 3.1. Chronic Migraineurs

The migraine frequency (F_3.545, 336.732_ = 109.446; *p* < 0.001), intensity (F_3.803, 361.285_ = 57.307; *p* < 0.001), and symptomatic medication intake (F_1.645, 148.073_ = 20.025; *p* < 0.001) significantly improved. There was no statistically significant difference between the three groups in terms of frequency (F_2, 95_ =0.539; *p* = 0.585), intensity (F_2, 95_ = 1.023; *p* = 0.364), or symptomatic medication intake (F_2, 90_ = 1.881; *p* = 0.152).

**Figure 2:**
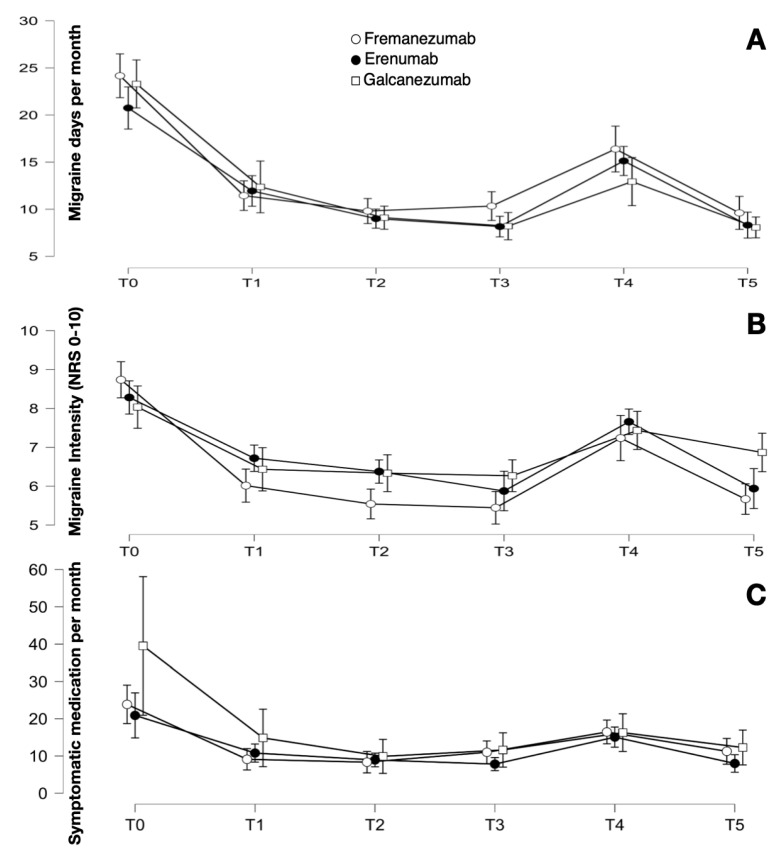
(**A**) Number of mean migraine days per month at the different phases of follow-up in chronic migraineurs, divided per anti-CGRP mAb. (**B**,**C**) Mean intensity and mean number of symptomatic medication intake per month, respectively. The bars represent the confidence interval.

See Table 3 and Figure 2 for detailed data.

In all three groups, there was a significant reduction in migraine frequency between baseline and T1 (*p* < 0.001 in all the groups), T2 (*p* < 0.001 in all the groups), T3 (*p* < 0.001 in all the groups), T4 (*p* < 0.001 in all the groups), and T5 follow-up (*p* < 0.001 in all the groups). There was a significant difference in the three anti-CGRP mAbs between the T3 and T4 (*p* < 0.001 erenumab and fremanezumab; 0.038 galcanezumab) as well as for T4 and T5 follow-up (*p* < 0.001 erenumab and fremanezumab; 0.026 galcanezumab). There was no statistically significant difference in migraine frequency comparing T3 vs. T5 in each group (*p* = 1.000), T1 vs. T2 (*p* = 1.000), T1 vs. T3 (*p* = 1.000 for fremanezumab, 0.302 for erenumab, 0.162 for galcanezumab), or T2 vs. T3 (*p* = 1.000).

Intensity improved in all three groups when comparing T0 to T1 (*p* < 0.001), T2 (*p* < 0.001), T3 (*p* < 0.001), and T5 (*p* < 0.001 for fremanezumab and erenumab, 0.043 for galcanezumab). The fremanezumab group had significantly lower intensity at T4 when compared to T0 (*p* < 0.001), while in the other two groups, this difference was not significant (*p* = 1.000). Moreover, there was a significant difference in terms of intensity between T3 and T4 in all three groups (*p* < 0.001 for fremanezumab and galcanezumab, 0.043 for galcanezumab). When comparing T4 vs. T5, there was a significant difference in the fremanezumab and erenumab groups, but not in the galcanezumab group (*p* < 0.001 fremanezumab and erenumab; 1.000 galcanezumab). T3 was not different from T5 in the three groups (*p* = 1.000). There were no differences between T1 vs. T2 (*p* = 1.000), T1 vs. T3 (*p* = 1.000 for fremanezumab and galcanezumab, 0.694 for erenumab), or T2 vs. T3 (*p* = 1.000).

Symptomatic medication intake was reduced in the fremanezumab group when comparing T0 (*p* = 0.011) to T1 (*p* = 0.004) and T2 but not to T3 (*p* = 0.073), and T5 (*p* = 0.093). T0 vs. T4 was not significant (*p* = 1.000). In the erenumab group, there was no significant difference between T0 and T1 (*p* = 1.000), T2 (*p* = 0.424), T3 (*p* = 0.177), or T5 (*p* = 0.203). T0 vs. T4 showed no differences (*p* = 1.000). The galcanezumab group had a significant reduction from T0 (*p* < 0.001) to T1 (*p* < 0.001), T2 (*p* < 0.001), T3 (*p* < 0.001), T4 (*p* < 0.001), and T5 follow-up (*p* < 0.001). There was no difference in T3 vs. T4 (*p* = 1.000), T4 vs. T5 (*p* = 1.000), or T3 vs. T5 (*p* = 1.000) in the three groups. There were no differences between T1 vs. T2 (*p* = 1.000), T1 vs. T3 (*p* = 1.000), or T2 vs. T3 (*p* = 1.000).

### 3.2. High-Frequency Episodic Migraineurs

**Figure 3:**
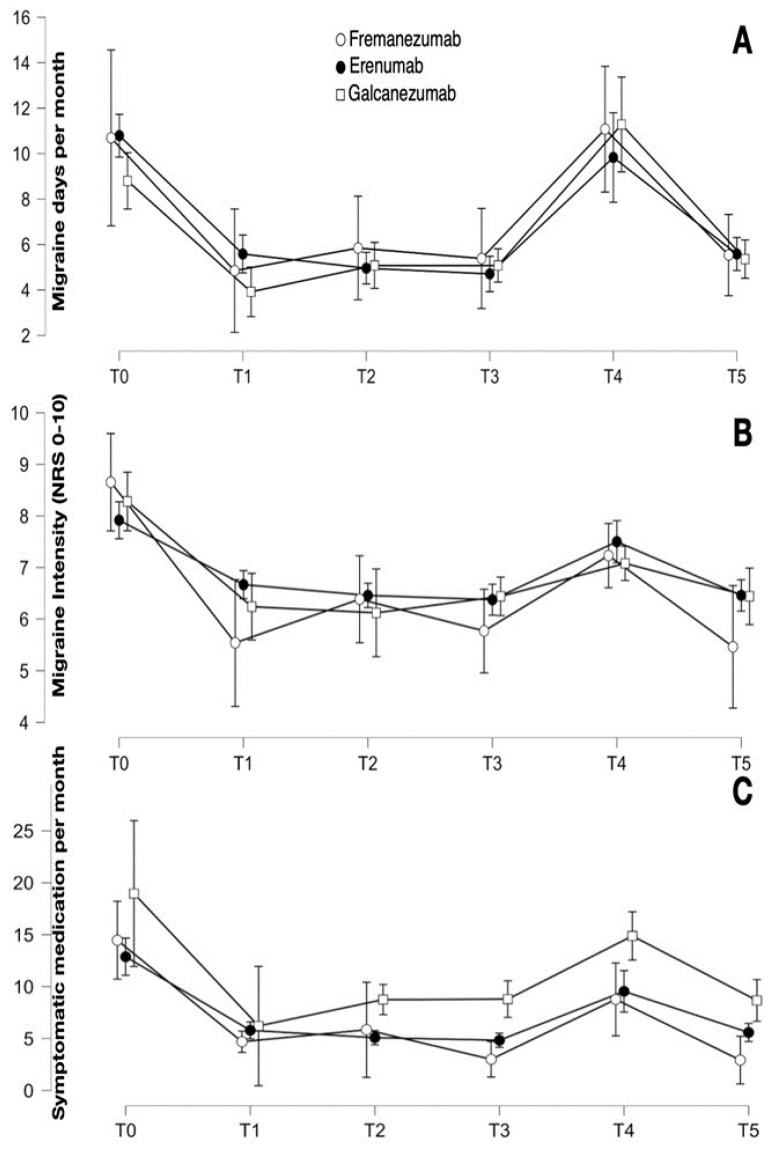
(**A**) Number of mean migraine days per month at the different phases of follow-up in high-frequency episodic migraineurs, divided per anti-CGRP mAb. (**B**,**C**) Mean intensity and mean number of symptomatic medications intake per month, respectively. The bars represent the confidence interval.

High-frequency episodic migraineurs improved in migraine frequency (F_2.894, 170.765_ = 41.063; *p* < 0.001), intensity (F_4.150, 244.838_ = 28.568; *p* < 0.001), and symptomatic medication intake (F_1.810, 104.963_ = 20.854; *p* < 0.001). There was no statistically significant difference between the three groups in terms of migraine frequency (F_2, 59_ = 0.203; *p* = 0.817), migraine intensity (F_2, 59_ = 0.513; *p* = 0.601), or symptomatic medication intake (F_2, 58_ = 1.641; *p* = 0.203). See Table 4 for detailed data and Figure 3.

The migraine frequency significantly improved from T0 to T1 (*p* < 0.001 fremanezumab; <0.001 erenumab; <0.001 galcanezumab), T2 (*p* = 0.015 fremanezumab; <0.001 erenumab; 0.006 galcanezumab), T3 (*p* = 0.004 fremanezumab; <0.001 erenumab; 0.006 galcanezumab), and T5 (*p* = 0.006 fremanezumab; <0.001 erenumab; 0.015 galcanezumab) follow-up in the three anti-CGRP mAbs, but not at T4 (*p* = 1.000 fremanezumab; 1.000 erenumab; 0.576 galcanezumab). In all three groups, the difference T3 vs. T4 (*p* = 0.001 fremanezumab, <0.001 erenumab, <0.001 galcanezumab), and T4 vs. T5 (*p* = 0.002 fremanezumab, <0.001 erenumab, <0.001 galcanezumab) was statistically significant. There was no statistical difference between T3 and T5 in any of the three anti-CGRP mAbs (*p* = 1.000 fremanezumab, 1.000 erenumab, 1.000 galcanezumab).

The three groups had significant reduction in migraine intensity from T0 to T1 (*p* < 0.001 fremanezumab and galcanezumab; 0.066 erenumab), T2 (*p* < 0.001 fremanezumab; 0.002 erenumab; <0.001 galcanezumab), T3 (*p* < 0.001 fremanezumab; 0.003 erenumab; <0.001 galcanezumab), and T5 (*p* < 0.001 fremanezumab; 0.008 erenumab; <0.001 galcanezumab); There was a worsening in migraine intensity at T4 that was not significantly different from T0 in all the three groups (*p* = 0.399 fremanezumab; 1.000 erenumab; 0.082 galcanezumab). Migraine intensity worsened at T4, but there was no statistical difference when compared to T3 (*p* = 0.317 fremanezumab; 0.206 erenumab; 1.000 galcanezumab) and T5 (*p* = 1.000 fremanezumab; 1.000 erenumab; 1.000 galcanezumab). T4 was statistically different from T5 on the fremanezumab group only (*p* = 0.040 fremanezumab; 0.416 erenumab; 1.000 galcanezumab).

The number of symptomatic medications per month significantly reduced from T0 to T1 (*p* = 0.033 fremanezumab; 0.040 erenumab; <0.001 galcanezumab), T2 (*p* = 0.154 fremanezumab; 0.010 erenumab; <0.001 galcanezumab), T3 (*p* = 0.003 fremanezumab; 0.006 erenumab; <0.001 galcanezumab), and T5 (*p* = 0.002 fremanezumab; 0.027 erenumab; <0.001 galcanezumab) in all the three groups. There were no differences between T0 and T4 (*p* = 1.000) in symptomatic medication intake in any of the three groups. Moreover, T3 was no different from T4 (*p* = 1.000 fremanezumab; 1.000 erenumab; 0.227 galcanezumab) and T5 (*p* = 1.000 in the three groups) in each group. T4 and T5 (*p* = 1.000 for fremanezumab and erenumab; 0.189 galcanezumab) were not statistically different either.

There were no differences between T1 vs. T2 (*p* = 1.000), T1 vs. T3 (*p* = 1.000), or T2 vs. T3 (*p* = 1.000) for the three variables.

There was no difference in response rate at different follow-ups among the three anti-CGRP mAbs. In chronic migraineurs, galcanezumab had a higher response rate during the 1-month suspension period when compared to erenumab and fremanezumab. In the high-frequency episodic migraineurs, fremanezumab had a higher response rate at 12-month follow-up. See Table 5 for the details.

There were 22 cases of constipation (3 with fremanezumab, 14 with erenumab, and 5 with galcanezumab). These cases were reported to be mild and presented during the entire follow-up. Seven patients reported localized skin reactions in the injection area shortly after the drug administration (two with fremanezumab, four with erenumab, and one with galcanezumab). These adverse events presented only once. There were no other adverse events.

## 4. Discussion

The three anti-CGRP mAbs effectively reduced migraine frequency, intensity, and symptomatic medication intake. There were no significant differences when comparing these data between the three anti-CGRP mAbs. The three anti-CGRP mAbs worsened in migraine frequency during the month of withdrawal from treatment, with resumption of efficacy upon restart. Chronic migraineurs improved similarly in terms of migraine days per month. In these patients, intensity also worsened at T4 in the three groups. The number of symptomatic medications increased at T4 in the three groups, but only the galcanezumab group still had a significantly lower number than baseline. Interestingly, T4 was not significantly different from T5 and T3. For high-frequency episodic migraine, the worsening during T4 determined a return to baseline for the migraine frequency, intensity, and symptomatic medication intake in the three groups. Moreover, at T5, there was a significant decrease in migraine frequency. Intensity improved only in the fremanezumab group, and the frequency of symptomatic medication intake per month was no different from T4 and T3.

Moreover, the response rate was also similar, with some differences: galcanezumab maintained a higher response rate during the suspension period than erenumab and fremanezumab. Interestingly, fremanezumab had a higher response rate at the 12-month follow-up; however, this result must be considered with caution, since the numerosity of the patients treated with fremanezumab is low.

Two recent meta-analyses, including 7 and 18 randomized controlled trials (RCTs), respectively, reported similar efficacy for the three anti-CGRP mAbs [15,16]. Two other meta-analyses involving 7 RTCs (3052 patients) and 11 RCTs (6397) reported that anti-CGRP mAbs that target the molecule were more effective than anti-CGRP mAbs that target the receptor [17,18]. Another meta-analysis, which analyzed 13 RTCs and evaluated the efficacy of onabotulinum toxin A, topiramate, and anti-CGRP mAbs, concluded that fremanezumab 675 mg in the first month and 225 mg in the second and third months were superior to the other therapies; moreover, erenumab 140 mg monthly was the most effective therapy in reducing symptomatic medication intake [19]. A systematic review analyzed episodic and chronic migraineurs treated with different preventive therapies (erenumab, fremanezumab, eptinezumab, galcanezumab, atogepant) or placebo. The most effective therapy in reducing migraine days per month was fremanezumab 225 mg [20]. These data appear contradictory, and this is to be expected since the population in the different studies is heterogeneous, and no direct comparisons were performed. Only a few real-world studies have compared galcanezumab, fremanezumab, and erenumab to prevent migraine, and none have compared the outcome during the suspension and subsequent reprisal.

Quintana and colleagues retrospectively analyzed 77 migraine patients, comparing galcanezumab, fremanezumab, and erenumab. Their results showed no statistically significant differences in the average number of migraine days per month, average monthly symptomatic medication intake, migraine disability assessment scale (MIDAS) score, and Headache Impact Test-6 questionnaires up to 6 months of follow-up [13].

In a prospective study involving 140 migraineurs (45 treated with erenumab, 54 with fremanezumab, and 41 with galcanezumab), the authors reported no differences in migraine frequency, MIDAS, or symptomatic medication intake. Interestingly, fremanezumab and galcanezumab in chronic migraineurs with MOH were superior to erenumab [21].

Similar results were found in a single-center Japanese real-life study, where no difference in monthly reduction in migraine days over 12 months was found between three CGRP mAbs [22]. Another study of 152 patients compared anti-CGRP mAbs that target the CGRP itself (68 patients; 49 galcanezumab, 19 fremanezumab) to erenumab (84 patients), which targets the CGRP receptor. There were no differences between the two groups in any of the variables analyzed. Interestingly, super-responders were more prevalent in the first group (galcanezumab and fremanezumab) [23].

An Italian multicentric observational study compared the response to anti-CGRP mAbs during the second year of therapy to the first year of treatment, and they did not find any differences in migraine frequency, symptomatic medication intake, or HIT-6. Moreover, they compared anti-CGRP against the molecule itself to anti-CGRP that targets the receptor (erenumab) and did not find any differences in terms of response during the first and second year of therapy; however, at baseline, the erenumab group presented higher migraine days per month [24].

A previous study revealed faster worsening in patients treated with erenumab than those treated with an anti-CGRP mAb after the first month of treatment cessation [25].

Nevertheless, whether the persistent benefit during the second year of treatment differs between the three types of antibodies is yet to be established. Our data reveal no significant differences in mean migraine days or attack intensity during drug discontinuation or in the first three months of the second year of resumption when comparing the three drugs.

### Limitations of the Study

Several factors limit the relevance of our results, first of which is the retrospective uncontrolled design of the study. In fact, the three groups had statistical differences in migraine frequency and intensity at baseline; however, our study showed that the response to the anti-CGRP mAbs was similar. Moreover, eptinezumab, erenumab 70 mg, and fremanezumab 675 mg every 3 months were not included. Finally, the sample was relatively small. Further studies with prospective controlled designs and larger samples are needed to confirm our observations.

## 5. Conclusions

Fremanezumab (225 mg), galcanezumab (120 mg), and erenumab (140 mg) demonstrated similar efficacy in reducing headache intensity, monthly headache days, and symptomatic medication intake. In the chronic migraine setting, galcanezumab showed a higher response rate during the 1-month suspension period when compared to erenumab and fremanezumab.

## Figures and Tables

**Table 1:** Demographics of each group. Quantitative data are reported as mean (SD); qualitative data are reported as frequencies. Legend: * group is statistically different vs. the other two groups; ** difference statistically significant vs. erenumab; *** difference statistically significant vs. galcanezumab.

	Fremanezumab	Erenumab	Galcanezumab	*p*
Number	49 patients	56 patients	55 patients	
Female sex	45/49 (92%)	49/56 (88%)	44/55 (80%)	0.285
Age	49.388 ± 11.729	46.661 ± 11.550	46.709 ± 13.536	0.443
Migraine onset	19.224 ± 12.256	19.582 ± 8.713	20.222 ± 11.423	0.893
Chronic migraine	36/49 (73%)	32/56 (57%)	30/55 (55%)	0.104
BMI	24.962 ± 5.236	24.047 ± 5.279	23.705 ± 4.113	
Migraine frequency (days per month)	20.592 ± 7.845 *	16.482 ± 6.427	16.891 ± 8.900	**0.015**
Intensity (NRS 1–10)	8.714 ± 1.056 *	8.125 ± 0.875	8.113 ± 1.171	**0.007**
Symptomatic medications intake (number per month)	21.128 ± 15.539	17.264 ± 14.836	30.941 ± 44.996	0.051
Previous prophylaxis	4.367 ± 1.424	4.250 ± 1.676	3.800 ± 1.568	0.146
Major depressive Disorder	16/49 (33%)	8/56 (14%)	10/55 (18%)	0.057
Familiarity for migraine	33/49 (67%)	36/56 (64%)	34/55 (62%)	0.895
Unilateral migraine	33/49 (67%)	41/56 73%)	34/55 (62%)	0.445
Aura	10/49 (20%)	9/56 (16%)	18/55 (33%)	0.100
Photophobia	42/49 (86%)	52/56 (93%)	47/55 (85%)	0.432
Phonophobia	41/49 (84%)	47/56 (84%)	43/55 (78%)	0.684
Osmophobia	26/49 (53%)	26/56 (46%)	25/55 (45%)	0.709
Nausea	45/49 (92%)	48/56 (86%)	47/55 (85%)	0.549
Vomit	25/49 (51%)	28/56 (50%)	22/55 (40%)	0.454
**Chronic Migraineurs**				
Migraine frequency (day per month)	24.167 ± 5.735 **	20.750 ± 5.187	23.300 ± 5.784	**0.039**
Migraine intensity (NRS 1–10)	8.736 ± 1.038	8.281 ± 1.054	8.033 ± 1.189 *	**0.032**
Symptomatic medication intake (number per month)	23.857 ± 16.914	20.897 ± 18.993	39.517 ± 53.425	0.076
**High-Frequency Episodic Migraineurs**				
Migraine frequency (day per month)	10.692 ± 2.394	10.792 ± 1.841 ***	8.800 ± 3.391	**0.024**
Migraine intensity (NRS 1–10)	8.654 ± 1.144	7.917 ± 0.504	8.280 ± 1.137	0.078
Symptomatic medication intake (number per month)	14.462 ± 7.195	12.875 ± 4.665	18.958 ± 26.795	0.545

**Table 2:** Data on the three groups regarding migraine frequency, intensity, and symptomatic medication intake at baseline (T0), 3-month follow-up (T1), 6-month follow-up (T2), 12-month follow-up (T3), 1-month suspension period (T4), and 3-month reprisal follow-up (T5). The data are presented as means ± SD.

Chronic and Episodic Migraine Patients (*n* = 160)	T0	T1	T2	T3	T4	T5
**Fremanezumab**						
Frequency (migraine days/month)	20.592 ± 7.845	9.694 ± 8.206	8.755 ± 8.092	9.020 ± 9.588	14.980 ± 9.660	8.531 ± 8.751
Migraine intensity	8.714 ± 1.056	5.888 ± 2.388	5.765 ± 2.280	5.531 ± 2.373	7.235 ± 1.945	5.612 ± 2.299
Symptomatic intake	21.128 ± 15.539	7.766 ± 9.037	7.532 ± 8.824	8.702 ± 14.859	14.043 ± 14.545	9.191 ± 15.905
**Erenumab**						
Frequency (migraine days/month)	16.482 ± 6.427	9.214 ± 7.032	7.268 ± 6.189	6.679 ± 5.970	12.857 ± 6.428	7.143 ± 6.297
Migraine intensity	8.125 ± 0.875	6.696 ± 1.320	6.411 ± 1.385	6.089 ± 1.654	7.589 ± 1.092	6.161 ± 1.511
Symptomatic intake	17.264 ± 14.836	8.528 ± 6.849	7.189 ± 6.251	6.472 ± 6.169	12.566 ± 8.780	6.906 ± 6.227
**Galcanezumab**						
Frequency (migraine days/month)	16.891 ± 8.900	8.527 ± 8.057	7.273 ± 5.626	6.782 ± 5.688	12.182 ± 7.493	6.836 ± 5.493
Migraine intensity	8.113 ± 1.171	6.264 ± 1.923	6.189 ± 2.001	6.358 ± 1.699	7.245 ± 1.479	6.679 ± 1.730
Symptomatic intake	30.941 ± 44.996	10.196 ± 13.862	9.686 ± 10.073	10.549 ± 14.036	16.059 ± 15.905	10.804 ± 14.058

**Table 3:** Group-wise data regarding migraine frequency, intensity, and symptomatic medication intake in chronic migraineurs at baseline (T0), 3-month follow-up (T1), 6-month follow-up (T2), 12-month follow-up (T3), 1-month suspension period (T4), and 3-month reprisal follow-up (T5). The data are presented as means ± SD.

Chronic Migraine	T0	T1	T2	T3	T4	T5
**Fremanezumab (36 patients)**						
Frequency (migraine days/month)	24.167 ± 5.735	11.444 ± 8.765	9.806 ± 8.078	10.333 ± 9.989	16.389 ± 19.123	9.611 ± 9.305
Migraine intensity	8.736 ± 1.038	6.014 ± 2.294	5.542 ± 2.278	5.444 ± 2.455	7.236 ± 2.079	5.667 ± 2.242
Symptomatic intake	23.857 ± 16.914	9.114 ± 10.070	8.343 ± 9.136	11.000 ± 16.700	16.457 ± 16.287	11.257 ± 16.814
**Erenumab (32 patients)**						
Frequency (migraine days/month)	20.750 ± 5.187	11.938 ± 7.947	9.000 ± 7.418	8.156 ± 7.274	15.125 ± 6.435	8.313 ± 7.826
Migraine intensity	8.281 ± 1.054	6.719 ± 2.294	6.375 ± 1.601	5.875 ± 1.913	7.656 ± 1.153	5.938 ± 1.777
Symptomatic intake	20.897 ± 18.993	10.793 ± 8.148	8.931 ± 7.625	7.828 ± 7.700	15.069 ± 10.299	8.000 ± 7.928
**Galcanezumab (30 patients)**						
Frequency (migraine days/month)	23.300 ± 5.784	12.367 ± 9.080	9.100 ± 6.697	8.200 ± 7.039	12.933 ± 8.170	8.067 ± 6.464
Migraine intensity	8.033 ± 1.189	6.433 ± 2.012	6.333 ± 1.971	6.267 ± 1.799	7.433 ± 1.501	6.867 ± 1.408
Symptomatic intake	39.517 ± 53.425	14.862 ± 19.639	9.897 ± 8.312	11.621 ± 15.129	16.276 ± 14.919	12.276 ± 15.026

**Table 4:** Data per group regarding migraine frequency, intensity, and symptomatic medication intake in high-frequency episodic migraine at baseline (T0), 3-month follow-up (T1), 6-month follow-up (T2), 12-month follow-up (T3), 1-month suspension period (T4), and 3-month reprisal follow-up (T5). The data are presented as means ± SD.

High-Frequency Episodic Migraine	T0	T1	T2	T3	T4	T5
**Fremanezumab (13 patients)**						
Frequency (migraine days/month)	10.692 ± 2.394	4.846 ± 3.262	5.846 ± 7.690	5.385 ± 7.567	11.077 ± 7.205	5.538 ± 6.671
Migraine intensity	8.654 ± 1.144	5.538 ± 2.696	6.385 ± 2.256	5.769 ± 2.204	7.231 ± 1.589	5.462 ± 2.537
Symptomatic intake	14.462 ± 7.195	4.692 ± 3.816	5.846 ± 7.777	3.000 ± 2.972	8.769 ± 5.790	2.923 ± 4.192
**Erenumab (24 patients)**						
Frequency (migraine days/month)	10.792 ± 1.841	5.583 ± 3.006	4.958 ± 2.774	4.708 ± 2.612	9.833 ± 5.130	5.583 ± 2.796
Migraine intensity	7.917 ± 0.504	6.667 ± 1.090	5.458 ± 1.062	6.375 ± 1.209	7.500 ± 1.022	6.458 ± 1.021
Symptomatic intake	12.875 ± 4.665	5.792 ± 3.310	5.083 ± 3.020	4.833 ± 2.959	9.542 ± 5.267	5.583 ± 2.796
**Galcanezumab (25 patients)**						
Frequency (migraine days/month)	8.800 ± 3.391	3.920 ± 2.431	5.080 ± 2.798	5.080 ± 2.722	11.280 ± 6.643	5.360 ± 3.639
Migraine intensity	8.280 ± 1.137	6.240 ± 1.921	6.120 ± 2.027	6.440 ± 1.557	7.080 ± 1.412	6.440 ± 2.043
Symptomatic intake	18.958 ± 26.795	6.208 ± 4.064	8.750 ± 11.895	8.792 ± 12.240	14.875 ± 17.038	8.667 ± 12.352

**Table 5:** Responder rate (>50% migraine days per month reduction) per group at the different follow-up periods.

50% Reduction in Migraine Days/Month	3-Month	6-Month	12-Month	1-Month Suspension	3-Month Reprisal
**Chronic and high-frequency episodic migraineurs**					
Fremanezumab	30/49 (61%)	40/49 (82%)	38/49 (78%)	16/49 (33%)	40/49 (82%)
Erenumab	30/56 (54%)	39/56 (70%)	40/56 (71%)	10/56 (18%)	36/56 (64%)
Galcanezumab	32/55 (58%)	37/55 (67%)	34/55 (62%)	18/55 (33%)	37/55 (67%)
Chi squared 𝜒^2^	0.642	3.033	3.138	4.018	4.245
*p*	0.726	0.219	0.208	0.134	0.120
**Chronic migraineurs**					
Fremanezumab	23/36 (64%)	29/36 (80%)	26/36 (72%)	14/36 (39%)	29/36 (80%)
Erenumab	16/32 (50%)	24/32 (75%)	24/32 (75%)	6/32 (17%)	23/32 (72%)
Galcanezumab	17/30 (57%)	23/30(77%)	23/30 (77%)	17/30 (57%)	23/30 (77%)
Chi squared	1.338	0.320	0.067	9.504	0.711
*p*	0.512	0.852	0.967	**0.009**	0.701
**High-Frequency Episodic Migraineurs**					
Fremanezumab	7/13 (54%)	11/13 (85%)	12/13 (92%)	2/13 (15%)	11/13 (85%)
Erenumab	14/24 (58%)	15/24 (63%)	16/24 (67%)	4/24 (17%)	13/24 (54%)
Galcanezumab	15/25 (60%)	14/25 (56%)	12/25 (48%)	1/25 (4%)	14/25 (56%)
Chi squared	0.134	3.129	7.413	2.237	3.789
*p*	0.935	0.209	**0.025**	0.327	0.150

## Data Availability

Data are contained within the article.

## Abbreviations

CGRP: calcitonin gene-receptor peptide; mAbs: monoclonal antibodies; RCTs: randomized controlled trials; MIDAS: migraine disability assessment scale; HIT-6: headache impact test 6; MOH: medication overuse headache.

## References

[1] Wattiez A.-S., Sowers L.P., Russo A.F. (2020). Calcitonin Gene-Related Peptide (CGRP): Role in Migraine Pathophysiology and Therapeutic Targeting. Expert Opin. Ther. Targets.

[2] Iyengar S., Johnson K.W., Ossipov M.H., Aurora S.K. (2019). CGRP and the Trigeminal System in Migraine. Headache.

[3] Al-Hassany L., Goadsby P.J., Danser A.H.J., MaassenVanDenBrink A. (2022). Calcitonin Gene-Related Peptide-Targeting Drugs for Migraine: How Pharmacology Might Inform Treatment Decisions. Lancet Neurol..

[4] Goadsby P.J., Edvinsson L., Ekman R. (1990). Vasoactive Peptide Release in the Extracerebral Circulation of Humans during Migraine Headache. Ann. Neurol..

[5] Goadsby P.J., Edvinsson L. (1993). The Trigeminovascular System and Migraine: Studies Characterizing Cerebrovascular and Neuropeptide Changes Seen in Humans and Cats. Ann. Neurol..

[6] Hansen J.M., Hauge A.W., Olesen J., Ashina M. (2010). Calcitonin Gene-Related Peptide Triggers Migraine-like Attacks in Patients with Migraine with Aura. Cephalalgia.

[7] Tepper S., Ashina M., Reuter U., Brandes J.L., Doležil D., Silberstein S., Winner P., Leonardi D., Mikol D., Lenz R. (2017). Safety and Efficacy of Erenumab for Preventive Treatment of Chronic Migraine: A Randomised, Double-Blind, Placebo-Controlled Phase 2 Trial. Lancet Neurol..

[8] Skljarevski V., Matharu M., Millen B.A., Ossipov M.H., Kim B.-K., Yang J.Y. (2018). Efficacy and Safety of Galcanezumab for the Prevention of Episodic Migraine: Results of the EVOLVE-2 Phase 3 Randomized Controlled Clinical Trial. Cephalalgia.

[9] Ferrari M.D., Diener H.C., Ning X., Galic M., Cohen J.M., Yang R., Mueller M., Ahn A.H., Schwartz Y.C., Grozinski-Wolff M. (2019). Fremanezumab versus Placebo for Migraine Prevention in Patients with Documented Failure to up to Four Migraine Preventive Medication Classes (FOCUS): A Randomised, Double-Blind, Placebo-Controlled, Phase 3b Trial. Lancet.

[10] Vernieri F., Altamura C., Brunelli N., Costa C.M., Aurilia C., Egeo G., Fofi L., Favoni V., Pierangeli G., Lovati C. (2021). Galcanezumab for the Prevention of High Frequency Episodic and Chronic Migraine in Real Life in Italy: A Multicenter Prospective Cohort Study (the GARLIT Study). J. Headache Pain.

[11] Robblee J., Devick K.L., Mendez N., Potter J., Slonaker J., Starling A.J. (2020). Real-World Patient Experience With Erenumab for the Preventive Treatment of Migraine. Headache.

[12] Caronna E., Gallardo V.J., Alpuente A., Torres-Ferrus M., Pozo-Rosich P. (2021). Anti-CGRP Monoclonal Antibodies in Chronic Migraine with Medication Overuse: Real-Life Effectiveness and Predictors of Response at 6 Months. J. Headache Pain.

[13] Quintana S., Russo M., Manzoni G.C., Torelli P. (2022). Comparison Study between Erenumab, Fremanezumab, and Galcanezumab in the Preventive Treatment of High Frequency Episodic Migraine and Chronic Migraine. Neurol. Sci. Off. J. Ital. Neurol. Soc. Ital. Soc. Clin. Neurophysiol..

[14] Headache Classification Committee of the International Headache Society (IHS) (2018). The International Classification of Headache Disorders, 3rd Edition. Cephalalgia.

[15] Soni P., Chawla E. (2021). Efficacy and Safety of Anti-Calcitonin Gene-Related Peptide Monoclonal Antibodies for Treatment of Chronic Migraine: A Systematic Review and Network Meta-Analysis. Clin. Neurol. Neurosurg..

[16] Wang X., Chen Y., Song J., You C. (2021). Efficacy and Safety of Monoclonal Antibody Against Calcitonin Gene-Related Peptide or Its Receptor for Migraine: A Systematic Review and Network Meta-Analysis. Front. Pharmacol..

[17] Wang X., Wen D., He Q., You C., Ma L. (2022). Efficacy and Safety of Monoclonal Antibody against Calcitonin Gene-Related Peptide or Its Receptor for Migraine Patients with Prior Preventive Treatment Failure: A Network Meta-Analysis. J. Headache Pain.

[18] Shi M., Guo J., Li Z., Sun H., Yang X., Yang D., Zhao H. (2021). Network Meta-Analysis on Efficacy and Safety of Different Anti-CGRP Monoclonal Antibody Regimens for Prophylaxis and Treatment of Episodic Migraine. Neurol. Res..

[19] Yang C.-P., Zeng B.-Y., Chang C.-M., Shih P.-H., Yang C.-C., Tseng P.-T., Wang S.-J. (2021). Comparative Effectiveness and Tolerability of the Pharmacology of Monoclonal Antibodies Targeting the Calcitonin Gene-Related Peptide and Its Receptor for the Prevention of Chronic Migraine: A Network Meta-Analysis of Randomized Controlled Trials. Neurother. J. Am. Soc. Exp. Neurother..

[20] Sun W., Cheng H., Xia B., Liu X., Li Y., Wang X., Liu C. (2023). Comparative Efficacy and Safety of Five Anti-Calcitonin Gene-Related Peptide Agents for Migraine Prevention: A Network Meta-Analysis. Clin. J. Pain.

[21] Saccà F., Braca S., Sansone M., Miele A., Stornaiuolo A., De Simone R., Russo C.V. (2023). A Head-to-Head Observational Cohort Study on the Efficacy and Safety of Monoclonal Antibodies against Calcitonin Gene-Related Peptide for Chronic and Episodic Migraine. Headache.

[22] Suzuki K., Suzuki S., Shiina T., Tatsumoto M., Fujita H., Haruyama Y., Hirata K. (2023). Effectiveness of Three Calcitonin Gene-Related Peptide Monoclonal Antibodies for Migraine: A 12-Month, Single-Center, Observational Real-World Study in Japan. Cephalalgia.

[23] Schiano di Cola F., Bolchini M., Ceccardi G., Caratozzolo S., Liberini P., Rao R., Padovani A. (2023). An Observational Study on Monoclonal Antibodies against Calcitonin-Gene-Related Peptide and Its Receptor. Eur. J. Neurol..

[24] Vernieri F., Brunelli N., Guerzoni S., Iannone L.F., Baraldi C., Rao R., Schiano di Cola F., Ornello R., Cevoli S., Lovati C. (2023). Retreating Migraine Patients in the Second Year with Monoclonal Antibodies Anti-CGRP Pathway: The Multicenter Prospective Cohort RE-DO Study. J. Neurol..

[25] Raffaelli B., Terhart M., Overeem L.H., Mecklenburg J., Neeb L., Steinicke M., Reuter U. (2022). Migraine Evolution after the Cessation of CGRP(-Receptor) Antibody Prophylaxis: A Prospective, Longitudinal Cohort Study. Cephalalgia.

